# The Potential of Digital Screening Tools for Childhood ADHD in School Environments: A Preliminary Study

**DOI:** 10.3390/healthcare11202795

**Published:** 2023-10-22

**Authors:** Ana-Marta Gabaldón-Pérez, María-Luisa Martín-Ruiz, Fernando Díez-Muñoz, María Dolón-Poza, Nuria Máximo-Bocanegra, Iván Pau de la Cruz

**Affiliations:** 1 Grupo de Investigación Innovación Tecnológica para las Personas (InnoTep), Departamento de Ingeniería Telemática y Electrónica, ETSIS de Telecomunicación, Campus Sur, Universidad Politécnica de Madrid, 28031 Madrid, Spain; a.gabaldon@upm.es (A.-M.G.-P.); marialuisa.martinr@upm.es (M.-L.M.-R.); fernando.diez.munoz@upm.es (F.D.-M.); maria.dolonp@upm.es (M.D.-P.); ivan.pau@upm.es (I.P.d.l.C.); 2 Department of Physiotherapy, Occupational Therapy, Rehabilitation, and Physical Medicine, Faculty of Health Sciences, Rey Juan Carlos University, 28922 Madrid, Spain

**Keywords:** attention deficit hyperactivity disorder, e-health, school, executive functions, screening tool

## Abstract

Attention deficit hyperactivity disorder (ADHD) is a highly prevalent developmental disorder in children. However, accurately identifying ADHD in early childhood remains a crucial challenge. Electronic health (e-health) systems offer promising possibilities to enhance the diagnostic process for ADHD, particularly concerning the executive functions (EFs) that play a direct role. This study aims to validate an evidence-based tool for screening ADHD through EFs in the school environment. The tool, named Sendero Gris, is designed for tablet devices and is based on a previously validated test with the same name. To ensure its validity, a comparison was made between the results obtained from the tool to be validated and the original format of the test. The analysis revealed no statistically significant differences between the two approaches at a 90% confidence level (*p*-value = 0.49). Moreover, a user experience study focusing on usability was conducted to assess the children’s inclination to use the developed tool, yielding highly positive results. The implementation of *Sendero Gris* on a tablet device, with its objective and versatile nature, seems to maintain the potential of the original format as a screening tool for ADHD.

## 1. Introduction

### 1.1. Background

#### 1.1.1. ADHD and ENFEN Tests

ADHD (attention deficit hyperactivity disorder) is defined as “a neurobiologically-based disorder, originating in childhood and with significant functional implications across various domains of an individual’s life”, involving a “pattern of inattention and/or hyperactivity/impulsivity, which interferes with the person’s normal functioning” [1]. The most classical approach to diagnosis is based on behavioral criteria, in which children’s behavior is assessed through surveys and interviews with the children themselves and with the people closest to them in their daily life activities, such as teachers and family members. In addition, no specific test can diagnose ADHD, and the DSM-5 requires the presence of a sufficient number of core symptoms and functional impairment [2]. This approach has some shortcomings related mainly to the introduction of biases in the evaluation, as well as the low specificity of the diagnosis [3,4].

Recent research has placed particular emphasis on two aspects of ADHD functioning: executive functions (EFs) and motivational processes [5]. There has been sufficient interest in EFs and motivational mechanisms for serious consideration of whether they have value in the diagnosis of ADHD [6]. The prefrontal area of the brain houses the EFs, which regulate higher mental processes, behavioral programming, problem-solving, and decision-making. Certain subfunctions of the supramodal function, such as sustained attention, mental flexibility, thought organization, and working memory, may be impaired in individuals suffering from ADHD [7]. There are several advantages of this type of approach over behavior-based assessments. First, if EF assessments are properly designed and validated, more objective results are obtained because the subjective component of the initial interpretation of the specialist and the rest of the community (families, educators, etc.) is reduced. In addition, at the operational level, the underlying cognitive processes can be better understood, obtaining more specific knowledge of the deficits that contribute to ADHD symptoms and favoring more personalized interventions (for example, working memory or inhibitory control can be strengthened depending on the child) in clinical, school, and even home settings using technologies such as serious games [8,9,10,11].

The Neuropsychological Evaluation of Executive Functions in Children (ENFEN) test battery [8,12], known as Evaluación Neuropsicológica de las Funciones Ejecutivas en Niños in Spanish, is a set of tests specifically designed to assess the maturity level of EFs in children which is widely used in Spain for ADHD diagnosis. ENFEN tests consist of a total of four paper-based tests: (1) Fluency, consisting of two tests of phonological fluency and semantic fluency, assessing working memory, verbal memory, etc.; (2) Trails, composed by a Grey Trail (implemented in this work) and a Color Trail, evaluate prediction ability, prospective memory, etc., based on the trail making test task [13]; (3) *Rings* measures the behavior programming, spatial orientation, etc.; and (4) Interference, which assesses the inhibitory capacity and mental flexibility [12].

In the ENFEN methodology, children perform each of these tests on paper under the supervision of a specialist, who monitors the collection of various parameters of information: task completion time, number of errors, etc. Once all of the tests in the battery are completed, the score obtained is calculated as a weighted measure of the different scores. In this way, a generic explanatory profile of EF performance is obtained, which assists the expert in the diagnostic process in an objective manner. As Navarro-Soria et al. concluded in 2020 [8], the ENFEN tool can be valued as an appropriate psychometric tool in the psychoeducational field, helping professionals in a school environment to be more aware of the areas of cognitive development in which a child diagnosed with ADHD will have more difficulties.

#### 1.1.2. ADHD and e-Health

The term electronic health (e-health) refers to the utilization of web-based systems and processes to enhance healthcare delivery, improve patient engagement, and enhance the overall healthcare experience [14]. E-health applications are increasingly being accepted in homecare and used in professional healthcare [15,16,17,18]. In other words, they are ubiquitous systems that enable patients to access various health-related actions without the necessity of visiting a healthcare center or always having a specialist physically present.

In 2016, Olsen et al. conducted a systematic review of the applications of e-health technologies in the context of ADHD [19]. Upon reviewing the article, it is notable that most of the studies included in this review focus on the utilization of e-health technologies for the assessment and management of various aspects related to the treatment of ADHD. Overall, these studies have demonstrated promising results. Numerous other papers, focusing on the utilization of such technologies for addressing the deficit following diagnosis, can be found in [20,21,22,23,24,25,26].

In relation to the utilization of these technologies in the domain of the diagnosis of neurodevelopmental pathologies in childhood, Reese et al. in their study related to the diagnosis of autism demonstrate that there exist minimal disparities in diagnostic accuracy between these types of technologies and standard procedures [27]. Moreover, both experts and patients’ relatives exhibit a preference for the adoption of such systems [27,28]. Concerning ADHD, only one study introducing e-health systems into its diagnostic process has been identified. Kemppinen et al. developed a clinical decision support system (CDSS) specially designed for adults, which facilitates a novel assessment of patients encompassing both diagnostic and treatment processes [29]. Furthermore, this CDSS ensures that the multidisciplinary team adopts a comprehensive and algorithmic approach to the treatment of adult patients with a confirmed diagnosis. Currently, e-health systems have proven their effectiveness in developing and enhancing skills affected by attention and hyperactivity deficits, while providing individuals with unlimited access in time and space to their therapeutic process [19].

Considering the aforementioned points, it is evident that there is a lack of development in e-health-based solutions, despite the proven efficacy of such systems in diagnosing various physical conditions. There is a clear need for solutions that facilitate access to discriminative tests for ADHD and that enhance the overall experience for the individual undergoing the assessment.

### 1.2. Goal of This Study

Attention deficit hyperactivity disorder is a neurodevelopmental disorder that globally affects 5–7.2% of youth and 2.5–6.7% of adults. Recent estimates indicate that its prevalence is even higher in children in the United States (U.S.), around 8.7% or 5.3 million [30]. To date, the diagnostic process for ADHD worldwide is based on a comprehensive multidisciplinary assessment, which determines whether the patient meets the diagnostic criteria of the DSM-5, alongside interviews with the patient, their family members, and their educators [31,32,33,34]. However, this approach, always requiring the involvement of a specialist and the child’s community (e.g., parents or teachers), is not devoid of subjective criteria, as the expert’s diagnosis relies on their personal observations of all the actors involved (even experts may have some bias, often caused by limitations in the resources needed to perform assessments in large groups) [35]. In addition, behavior-based diagnoses do not provide clues to help target therapeutic treatments more precisely to specific aspects of the deficit. Improving the accuracy of ADHD diagnostic procedures will assist clinicians in designing more effective therapeutic approaches and facilitate the child’s participation in natural settings.

Currently, there are validated test batteries that can be useful in supporting the diagnostic process of ADHD in children aged 6 and above with approaches other than behavioral. This is the case for the ENFEN test [8,12]. This test assesses the neuropsychological maturity of individuals by analyzing various sub-functions, called executive functions, providing specialists with an objective source of support for diagnosing the specific deficit. This approach avoids existing biases in behavioral approaches, in addition to offering more detailed information on the most relevant aspects of the child’s deficit, and can better guide therapeutic actions.

However, despite the progress made in the assessment of EFs, the existing tests for this type of assessment, such as the aforementioned ENFEN test, have constraints affecting the overall performance of the assessment.

Because the child must travel to the assessment center, they are exposed to an environment in which they may not feel comfortable, which affects their natural performance [35]. In addition, the mandatory presence of a specialist to supervise the performance of the test gives rise to two new difficulties. On the one hand, given that the number of specialists is limited and that the protocol requires being aware of each child taking the test, screening procedures for large groups, such as those formed in schools, are difficult to manage in a reasonable amount of time. On the other hand, the specialist must make real-time decisions about the child’s performance during the observation, a process that is prone to errors and biases that affect the effectiveness of the tests.

The primary aim of this study is to conduct a comparative analysis of the discriminative power in assessing attention deficit hyperactivity disorder (ADHD) using the same test administered in two different formats: paper and digital via tablets. The test, known as “Sendero Gris”, is derived from the previously validated EF-based ENFEN test battery. The paper-based test is in the traditional and currently validated format within the ENFEN protocols and requires the presence of a specialist during administration. The tablet-based format, which does not require the presence of a specialist while children complete the test, has not been validated thus far.

The comparative study evaluates whether the test in tablet format can achieve a level of discrimination equal to (or higher than) the paper-based screening method in school environments and overcome the limitations of paper-based tests.

In addition to the indicated study, the article shows the main steps of the test construction process, indicating technical details to help in the replication of this type of solution for other areas or tests.

## 2. Materials and Methods

### 2.1. Application Framework

As mentioned earlier, this paper describes the implementation process of one of the ENFEN screening tests originally performed on paper, *Sendero Gris* (Grey Trail), on digital devices.

The chosen technological device to implement the solution was a tablet. Among other advantages, this type of device offers the benefit of being familiar to today’s children and has the capacity to collect relevant and diverse information parameters, such as the time between events and the number of touches on the screen [36]. The solution was implemented using the Kodular platform, which is a programming environment that enables the development of block-based coding applications for Android devices [37].

**Figure 1:**
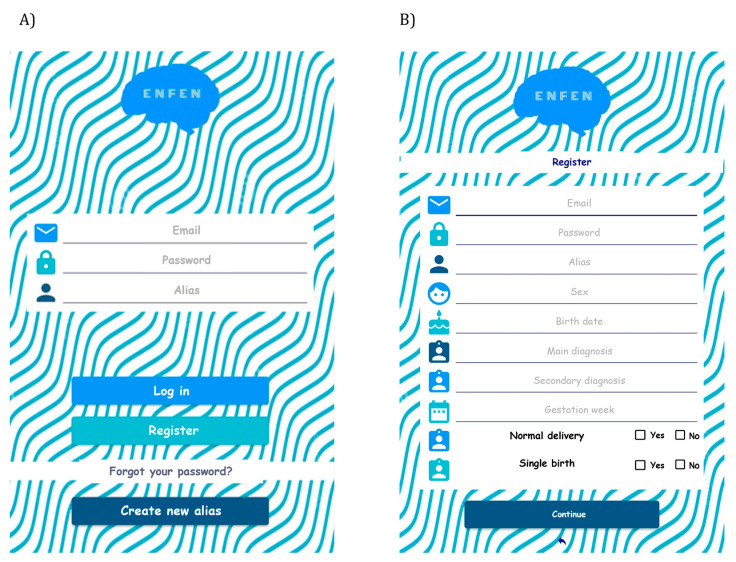
ENFEN application: (**A**) login screen and (**B**) first registration screen.

**Figure 2:**
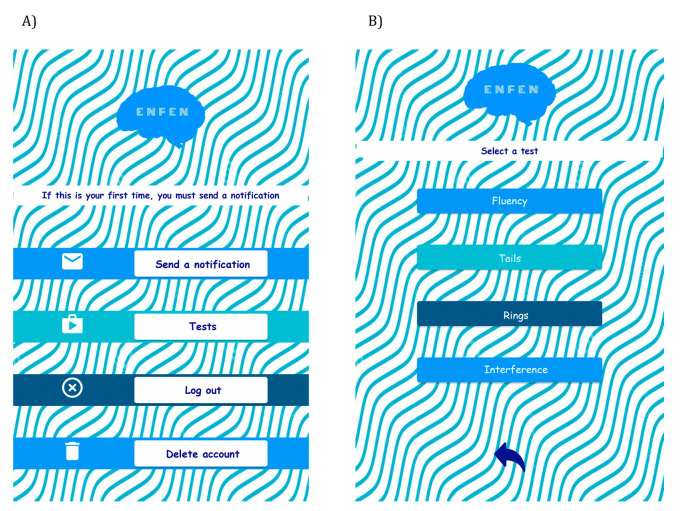
ENFEN application: (**A**) main menu and (**B**) test selection screen.

The developed app serves as a test container and currently only has *Sendero Gris* fully implemented within the app (Figure 1 and Figure 2).

The operation of the application, which aligns with the design and protocol of the validated ENFEN paper-based test, is as follows. Once the application is started, the app logo is displayed, followed by the login screen (Figure 1A). If it is the user’s first time accessing the app, they will be redirected to the registration screen (Figure 1B). Returning users can simply enter their email and password to access the tests. Figure 1A includes an option for an alias, allowing the same user (with the same registration email) to use different names for self-assessment. This eliminates the need for multiple user accounts for children within the same school or family; instead, using different aliases for each child is sufficient. Additionally, as shown in Figure 1B, certain data and control questions (e.g., sex, gestation weeks, main previous diagnosis if any, etc.) are requested, which are crucial for establishing connections between collected information and ensuring accurate diagnosis by the specialist.

Once the user has completed the registration process, a verification email will be sent to the provided email address. After verification, the user will gain access to the main menu of the application (Figure 2A). From this screen, the user can report errors and access the different tests, among other options. Finally, Figure 2B displays the selection screen for the available tests.

**Figure 3:**
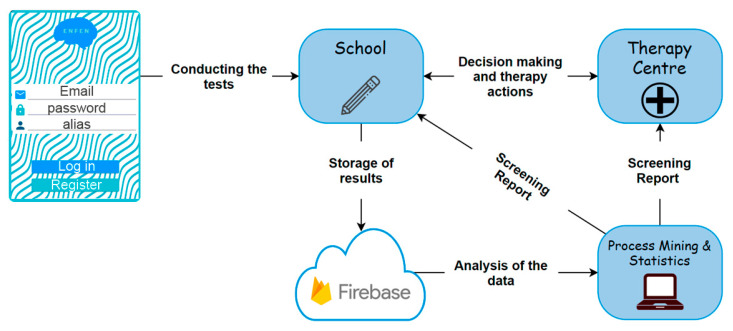
Outline of the ENFEN application.

Figure 3 illustrates the general functioning of the system. When the user interacts with the application, the personal data, control questions, and test results obtained are sent to a Firebase database [38]. Access to this database is restricted to registered therapists, ensuring the privacy of all participants.

### 2.2. Test Design

**Figure 4:**
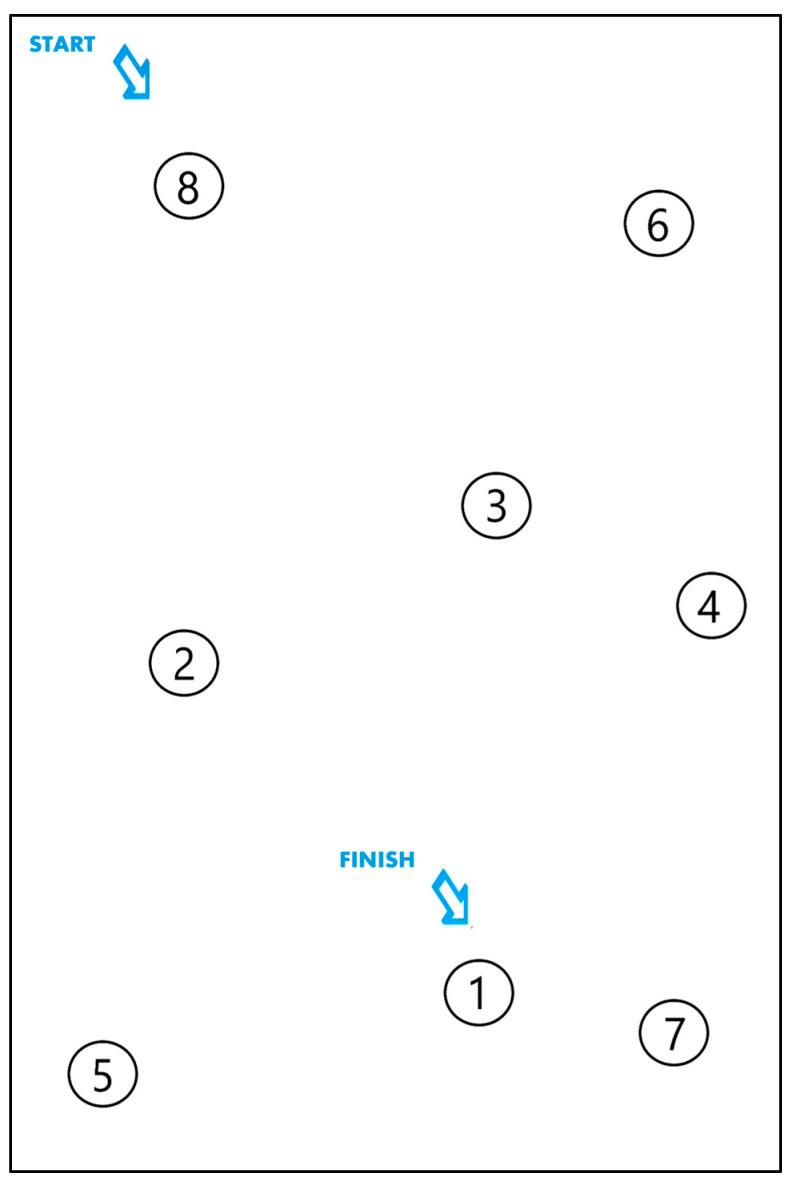
Sendero Gris from ENFEN. Training version.

The *Sendero Gris* test is designed to assess the ability to connect consecutive numbers, ranging from 1 to 8 in the training version and from 1 to 20 in the evaluation version (Figure 4).

The nodes, arranged randomly on the paper, need to be connected by a path or stroke in decreasing order to maximize the discriminative potential of the test. Among others, some of the sub-functions assessed in this test are sustained and selective attention, inhibitory control, working memory, and decision making.

The performance in the earlier sub-functions is measured based on the number of correct responses in the path of nodes drawn by the child (hits), errors in this path (substitutions), and omissions of nodes made by the child during the test, as well as the time required to complete it. The definition of these terms is detailed in the ENFEN manual [12].

Although the graphical construction of the application may appear simple (with a minimalist appearance), there are several fundamental aspects in its construction, referring to the collection of information, that cannot be overlooked (Table 1). In addition, to define solutions to the challenges presented in this table, several concepts related to the nodes had to be described:

- Start node: the node number from which the path is initiated.
- Target node: the node intentionally chosen as the next step in the path.
- Pressed nodes: the set of nodes collected by the application and considered for calculating the player’s final score.
- Final node: the node that contains the number 1. Once this node is considered a pressed node, the test is completed.

As the points listed in the previous table pertain to the data collection and calculation of the children’s final score, all these implementation aspects are crucial in the development of the solution, thus determining its effectiveness.

**Figure 5:**
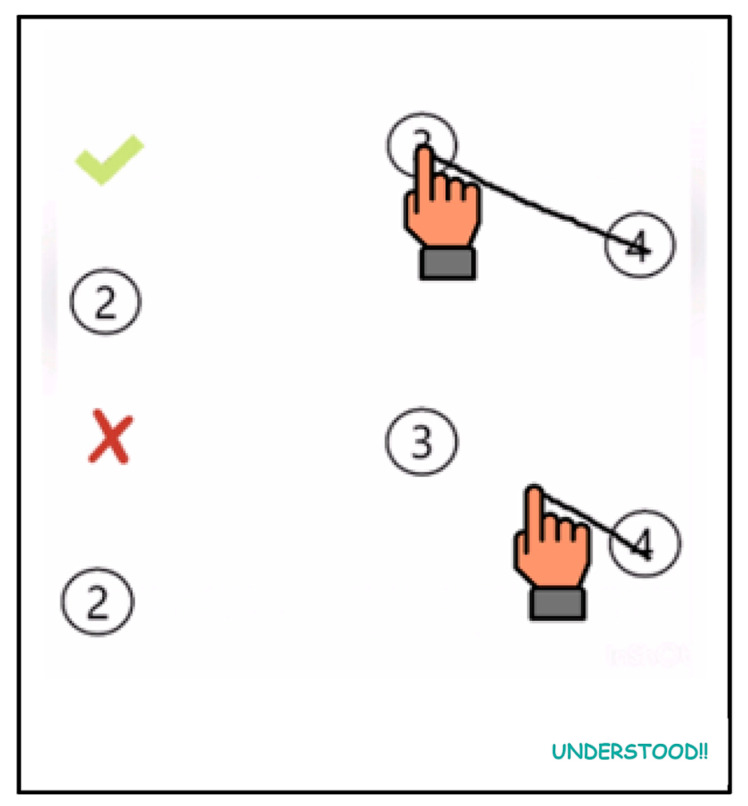
Explanatory GIF for linking the nodes.

Regarding passing over a node while moving towards the target node, the strategy adopted was to consider a node as pressed only when the child lifts their finger from the screen. This proper execution technique is demonstrated through a GIF animation after the instructions have been provided (Figure 5).

Regarding the measurement of the time taken by the child, the opportunity to communicate the rules was exploited. Therefore, once the individual has finished reading the guidelines, they should press the “understood” button to initiate the test, and the timer will start counting. Similarly, when the child presses the final node, the timer will stop.

In the case of the final node, the determination of whether it has been intentionally pressed is left to the player. That is, once the system recognizes that the number 1 has been pressed and the test can be considered complete, the timer stops, and a dialogue box appears asking if the child considers that the test has concluded. If the answer is no, the timer restarts, and the test continues as usual. On the other hand, if the individual responds in the affirmative, the test is over.

Concerning the explanation of the test rules, through a dialogue box and a voice message (auditory and visual aids) the child is informed about the required steps. This approach ensures that the test is accessible to a wider range of individuals [34].

Finally, in relation to measuring inactivity, specific periods of inactivity were established in collaboration with the experts. Consequently, periods of 15 s during which the tablet does not detect any interaction are considered as inactive. If this occurs, an informational message will be displayed on the screen for the child, who will have the option to either “continue playing” or “end the test”. These notifications will be displayed a total of three times. If 45 s of inactivity are detected, the test will automatically end, and the child will be assigned a predetermined maximum time.

Once all the technical characteristics necessary for the proper collection of the relevant markers have been specified, the method for calculating the final score obtained in the test is determined. By maintaining the concepts of hit, substitution, and omission according to their original definition in the ENFEN, the formula by which the test scores the child’s performance is presented in [12].

### 2.3. Hypothesis and Experimental Protocol

This study aims to test the hypothesis that there is no difference in the results between the paper-based *Sendero Gris* test, which is the original test, and its implementation as an e-health system (the tablet-based *Sendero Gris* test). If the hypothesis is accepted, it would indicate that the proposed e-health system has the same screening capability as the original test, under much higher conditions of comfort for the child, while eliminating bias in the assessor’s correction.

The experiments to test the hypothesis were undertaken in two scenarios: the first at the *Amanecer* school and the second at the *Centro Municipal de Asociaciones de Salud de Alcorcón*, both located in Madrid, Spain.

To assess the effectiveness of the proposed solution, a use case was designed to compare the results of the original paper-based test with those obtained using the tablet-based version. Thus, the same group of children participated in both versions of the test, including children with and without a diagnosis of ADHD.

Firstly, when taking the paper-based test, an expert was present to provide guidance to the children and supervise the test execution. The expert also recorded the time taken by the child to complete the test and calculated their final score according to the rules defined in the ENFEN protocol [12]. Once the paper version of the test was completed, for the tablet-based test, the child was provided with a preconfigured tablet that had the application installed, the user account logged in, and the control questions answered by their family members beforehand. No additional explanation about the test procedure or task was given to the child, and they simply started interacting with the tool and performed the test.

The experiment and subsequent study were conducted in accordance with the principles outlined in the Declaration of Helsinki and received approval from the Ethics Committee of Rey Juan Carlos University, with informed consent obtained from the families of the children who participated.

### 2.4. Sample Summary

Due to the limited availability of eligible participants for the study, the initial sample size consisted of 28 individuals. Unfortunately, due to data collection issues related to technical issues on one of the hardware devices (the storage system failed), not all data from 4 players could be retrieved after the experiment was run, resulting in a final effective sample size of 24 children.

The initial sample consisted of 28 children of both sexes (12 girls and 16 boys), ranging in age from 6 to 12 years old (average 9.21, SD 2.01). Table 2 provides an overview of the sample, including the number of children by age and sex. It can be observed that the sample covers all the age groups for which the ENFEN battery was developed.

Regarding the control questions collected in the initial application form (Figure 1B), the average gestational weeks is 39.39, with a minimum of 34 weeks and a maximum of 42 weeks. Approximately 36% of the mothers of the children in the sample reported experiencing some delivery complications, with the majority being induced cesarean sections. Among the children born with birth complications, 60% received a diagnosis of ADHD. Moreover, all cases included in the sample were single births.

Table 3 presents the distribution of children according to sex and their ADHD diagnosis. Among the girls, a total of 4 had a diagnosis of ADHD (33.33%), while the majority of the sample consisted of undiagnosed girls (66.66%). In the case of males, 62.5% had a diagnosis of the deficit in question.

Lastly, Table 4 summarizes some diagnostic parameters of interest for the experts. On average, for both sexes, children with ADHD are older than those undiagnosed. On the other hand, there does not seem to be a clear association between gestational weeks and the disorder, as the average gestational weeks for both groups coincide. Finally, regarding birth complications, it was observed that 3 out of 4 girls with ADHD were born without any complications (75%), while 50% of the boys with ADHD (5 out of 10 children) suffered birth complications.

### 2.5. Statistical Method

Two different statistical analyses were performed. On the one hand, a simple statistical comparison based on averages was made between the collected parameters of both the paper-based test and the tablet-based test. The parameters were described in the Materials and Methods section: success (hits), error (substitutions), omissions, time consumed, and final score obtained. On the other hand, the non-parametric Wilcoxon signed-rank test was employed to assess the statistical significance of the difference in mean scores obtained in each test format [39]. This test is a non-parametric statistical hypothesis test used to compare the locations of two populations using two paired samples. Moreover, it is applicable for hypothesis testing with sample sizes of at least 16 individuals.

## 3. Results

### Obtained Results

For the comparison between the application and the original test, the error measurement parameters (substitutions and/or omissions) and successes achieved by each child during their evaluation were utilized.

As commented on in the Methods section, due to issues with data collection, a total of four participants had to be excluded from the analysis (two from each diagnostic group).

In the case of the paper-based version, Table 5 displays negligible disparities in benchmark scores between children with ADHD and those without. Diagnosed individuals, with an average of 18.69 hits, are only 0.22 points behind the undiagnosed children. Furthermore, in terms of the total time taken to complete the paper-format test, a discrepancy of approximately 5 s can be observed between the two groups.

In the case of the tablet-based test, the disparities between the final scores for both groups are more pronounced compared to the previous scenario (Table 6). The undiagnosed group achieved an average of 18.75 hits per child, similar to the paper version, whereas the ADHD group attained an average of 16.84 hits. Simultaneously, the time difference between the two groups was approximately 8 s.

**Figure 6:**
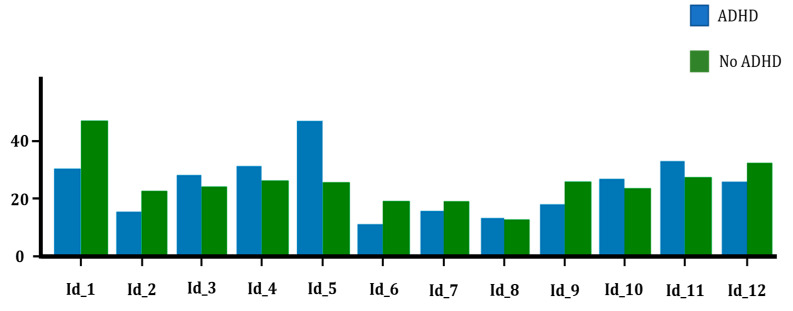
Final scores for the paper-based test.

**Figure 7:**
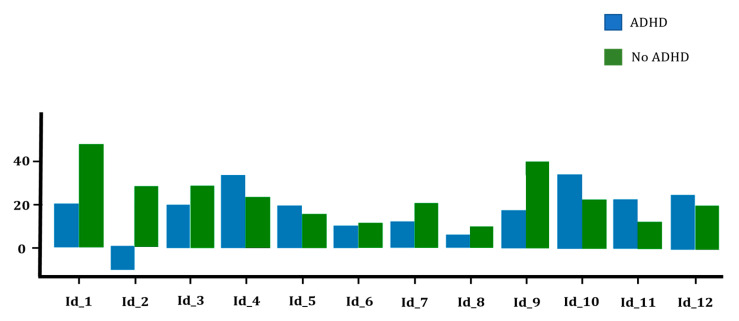
Final scores for the tablet-based test.

Now, when examining the individual scores obtained by each player in each of the tests (Figure 6 and Figure 7), we observed a negative final score for diagnosed children in the tablet-based version. This negative score is a result of one child connecting the nodes in an increasing order, from 1 to 20, right from the start of the test, contrary to the specified instructions to connect them in a decreasing order. Upon reviewing this child’s test on paper, it is impossible to determine, as there is no record of the direction in which the nodes were connected, whether the child executed it correctly (descending) or, due to human error, carried out the test in an ascending manner and it went unrecorded. Regardless, the presence of such a low score significantly reduces the average for the ADHD group compared to the opposite group.

Lastly, the Wilcoxon signed-rank test was applied. A value of W statistic = 219 was obtained (*p* = 0.49; alpha = 0.10). Thus, with a 90% confidence level, there is no statistical evidence to reject the hypothesis of equal results between the two tests.

**Figure 8:**
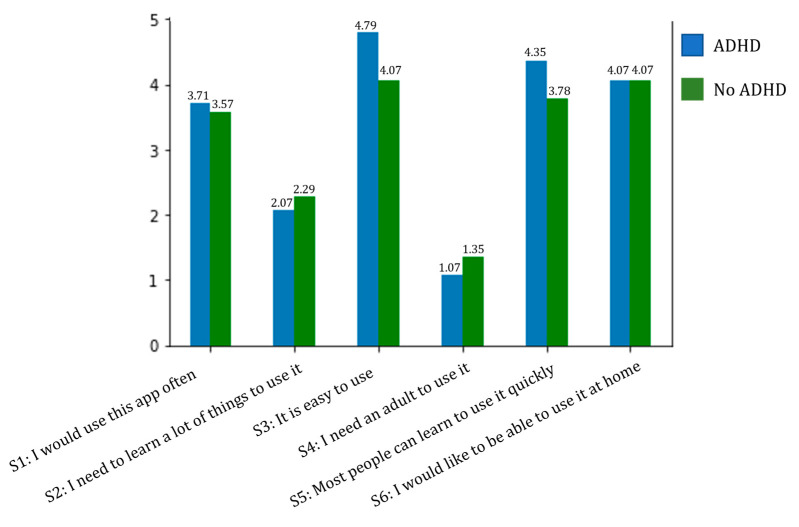
Results obtained for the user experience test and distributed according to whether the child had a diagnosis of ADHD.

Additionally, the users were provided with a user experience form, and qualified personnel (experts in usability) assisted them in filling it out. For a total of six statements, children had to rate (on a scale of 1 to 5) their intention to use the test and the difficulties they had found using the test on a tablet device. A rating of 1 represented “I strongly disagree with the statement”, while a rating of 5 represented “I strongly agree with the statement”. The results of the test are presented in Figure 8. Regarding statements S1 and S6, which assessed the intention for frequent use of the developed system, both groups exhibited mean scores above 3.5, with maximum values of 3.71 and 4.07, respectively. For S2 to S5, both groups exhibit very similar average scores, with a greater discrepancy in terms of game difficulty. For the group of children with ADHD, the game was found to be easier to use than for the control group, indicating a lower need for adult supervision.

## 4. Discussion

ADHD is recognized as one of the neuropsychological disorders that significantly impact the development and integration of individuals into society, “with well-established diagnostic and treatment services available throughout most of Europe” [40,41,42].

However, at present, screening related to this deficit, usually associated with a high number of children, lacks methods and tools that allow it to be carried out objectively and efficiently, which leads to an excessive burden both on specialists, who tend to assess in a biased way because they do not have the appropriate conditions, and on the children themselves, who must travel to specialized centers, which can affect their performance during the evaluation [34,43]. Fortunately, with advancements in technology and existing knowledge about ADHD, it is now possible to develop e-health-based solutions that facilitate the screening of the deficit for healthcare professionals. These solutions have the potential to improve the work of specialists and the quality of life for patients by providing accessible and efficient tools.

This article presents the findings of a comparative analysis aimed at assessing the discriminative efficacy of a validated ADHD test, known as Sendero Gris, and a component of the ENFEN test battery. The study involved administering the test in two formats: a traditional paper-based version, which is the format in which the test was designed, and a tablet-based version, both conducted within a school environment.

The results indicate that the tablet-based version of Sendero Gris has demonstrated promising discriminative power, comparable to the paper-based version. This encourages the research team to move forward with the implementation of all four ENFEN tests, aiming to create a comprehensive tool for screening ADHD in school-aged children.

One limitation of the present study is the relatively small sample size available. This limitation arose due to challenges in recruiting target individuals who were willing to participate in the study, as well as operational errors in implementing the proposed solution. As a result, the sample size was restricted to just 24 individuals. This constraint hinders our ability to generalize the findings on a broader scale, rendering this study a preliminary exploration of the discriminative potential of the proposed solution.

When comparing the scores obtained from the different test formats (supervised paper-based tests administered by specialists and unsupervised tablet-based tests), statistically similar results were observed. Furthermore, note that any learning that the children might have acquired from undertaking the test on two occasions is not a factor that warrants consideration as significant. In other words, if learning had occurred during the first instance of testing, improved performance could have been anticipated during the second iteration. As this is not the case and comparable outcomes were achieved in both instances, the exploration of the interaction stemming from the learning factor was deemed unnecessary.

Consequently, it can be inferred that the digitization of the ENFEN battery shows promise as a screening tool that is, at the very least, as effective as the original test while offering greater user convenience. It is essential to note that the Sendero Gris test exclusively assesses specific EFs. Therefore, not all individuals diagnosed with ADHD will necessarily display impairments in these particular EFs. Table 5 and Table 6 illustrate the test’s discriminative power in its two existing versions for identifying individuals with limitations in the assessed EFs. Furthermore, the manual calculation of scores by children has been removed, introducing an automated calculation process during the test’s execution.

In reference to statements S1 and S6, which assessed the intention of frequent usage of the developed system, it is noteworthy that the end users have exhibited a high degree of acceptance towards the solution. As for the statements addressing the test’s level of difficulty (S2 to S5), undoubtedly, overall, the participants found the test to be quite straightforward across both groups. This observation emphasizes that the presence of an adult during its execution was unnecessary.

Moreover, during the implementation of the presented study, other advantageous aspects of the digital format were discovered, such as the clear recording of the order in which the nodes are connected and the ability to collect a greater number of markers of interest compared to the current test.

However, the application did exhibit some functional errors and issues related to information collection. In certain instances, children had to be excluded from the analysis because of data collection failures while taking the test, which stemmed from errors in one of the hardware devices. Additionally, we identified instances of repeated traces, where the connection of two consecutive numbers occurred more than once resulting in error scores being added to their final score.

The gold standard for diagnosing ADHD is a specialized clinical evaluation that cannot be replaced. However, access to such assessments is limited in certain populations, especially without a proper screening process that allows for a more efficient allocation of existing resources [20]. Additionally, tests based on EFs not only facilitate the evaluation process but can also provide important clues for a more efficient diagnostic process by providing indications of specific deficits. Therefore, the presented solution has the potential to implement this screening process more efficiently, which, in turn, will allow us to focus more on existing resources for an accurate clinical diagnosis. Based on the results obtained, the researchers involved in this study are confident that the implementation of the complete set of ENFEN battery tests as an e-health system would greatly contribute to the effective and user-friendly detection of ADHD at an early age in natural settings.

## 5. Conclusions

ADHD affects all aspects of a human being, from their social life to personal and school development. Its onset is marked in childhood and can be diagnosed from the age of six. Therefore, an early and accurate diagnosis, followed by appropriate treatment, can mitigate and/or eliminate future challenges associated with the condition. This study, to our knowledge, is the first to introduce and validate the discriminatory ability of a digital screening tool to assess ADHD in children compared to a previously validated paper-based test. The tablet-based test addresses several significant limitations of the paper-based format. It can be conducted in a natural environment without the necessity for specialized supervision. Additionally, it enables objective tracking of a child’s progress throughout the test, thereby eliminating the need for mandatory specialist presence and the potential for observation bias. By addressing these limitations of paper-based tests, the developed solution offers an innovative approach to ADHD screening, leveraging e-health system technologies to enhance the whole process.

Finally, the potential for future enhancements includes the addition of more markers of interest, incorporating new tests, and exploring novel methods of analysis and result measurement. These advancements have the potential to further enhance the discriminatory power of the system.

## Figures and Tables

**Table 1:** Aspects to be implemented concerning data collection.

Implementation Aspects	Description
Area of the nodes	The node area should be adequately adjusted. Therefore, if a child is moving towards the target node and happens to pass close to another node while defining their path, the latter should not appear as pressed
Pass over a node	Among the rules, it is possible to pass over other nodes on the screen while moving towards the target node. In such cases, those numbers should not be considered as pressed nodes and therefore should not be included in the final result
Total time required	The time elapsed from when the player presses the “understood” button until they reach the final node
Test rules	The method for explaining the rules must be agreed upon with the specialist, as it is a crucial requirement
Measurement of inactivity	The test implementation aims to redirect the individual’s attention in case it detects a period of inactivity

**Table 2:** Number of children by age and sex.

	Age (Years)							
	6	7	8	9	10	11	12	Total
Female	2	2	1	1	3	1	2	12
Male	1	3	1	3	2	4	2	16
Total	3	5	2	4	5	5	4	28

**Table 3:** Number of children according to ADHD diagnosis and sex.

	ADHD Diagnosis		
	Yes	No	Total
Female	4	8	12
Male	10	6	16
Total	14	14	28

**Table 4:** Control questions conducted by diagnosis and sex.

	Age Avg.		Gestation Weeks Avg.		Normal Birth	
	ADHD	No ADHD	ADHD	No ADHD	ADHD	No ADHD
Female	9.25	8.87	39.25	39.25	3	7
Male	9.8	8.67	39.5	39.5	5	3

**Table 5:** Marks for the paper-based test.

	Hits Avg.	Substitutions Avg.	Omissions Avg.	Time Spent Avg. (s)	Final Score Avg.
ADHD	18.69	0.23	0.38	92.69	19.50
No ADHD	18.91	0.08	0.25	87.83	21.15

**Table 6:** Marks for the tablet-based test.

	Hits Avg.	Substitutions Avg.	Omissions Avg.	Time Spent Avg. (s)	Final Score Avg.
ADHD	16.84	2	0	108.76	13.64
No ADHD	18.75	0.17	0.08	100.83	18.34

## Data Availability

Restrictions apply to the availability of this data. The data were obtained from study participants with prior informed consent from their parents and are available by contacting the authors, with mandatory prior informed consent from the families involved, in compliance with the General Data Protection Regulation (Regulation (EU) 2016/679), and the development of a new Ethical Approval.
